# Time to Step-Up Our Game Concerning Nutrient Analysis of Pasteurized Donor Human Milk?

**DOI:** 10.1016/j.advnut.2024.100242

**Published:** 2024-05-30

**Authors:** Deborah L O’Connor

**Affiliations:** 1 Department of Nutritional Sciences, Temerty Faculty of Medicine, University of Toronto, Toronto, Ontario, Canada; 2 Translational Medicine Program, The Hospital for Sick Children, Toronto, Ontario, Canada; 3 Department of Pediatrics, Sinai Health, Toronto, Ontario, Canada

In recognition of the benefits of breastfeeding, the WHO, the American Academy of Pediatrics, the Dietary Guidelines for Americans, and Health Canada recommend exclusive breastfeeding for ∼6 mo after birth for healthy term infants [[1], [2], [3], [4]]. Health benefits include protection against child infections and malocclusion, a probable decrease in obesity and diabetes, and an increase in intelligence [4]. For preterm infants born of very low birth weight (VLBW; i.e., <1500 g), provision of human milk, specifically maternal milk with supplemental pasteurized donor milk (PDHM) (compared with infant formula) additionally reduces the risk of necrotizing enterocolitis (NEC), a potentially devastating gastrointestinal emergency [[5], [6], [7]]. As such, it is recommended that PDHM be fed to VLBW infants when mother’s milk is not available, insufficient, or contraindicated [8].

Although inequities in access to PDHM remain, a microbiologically safe supply of PDHM is available for VLBW infants born in North America through both nonprofit milk banks, primarily members of the Human Milk Banking Association of North America (HMBANA), and for-profit entities. HMBANA, like a number of its international counterparts (e.g., European Milk Bank Association) have independently developed and implemented guidelines for screening of milk donors and collection, processing, and distribution of PDHM [9,10]. Holder pasteurization (temperature held at 62.5°C for 30 min) is specifically used by HMBANA member banks to mitigate transmission of known pathogens through PDHM that may harm an infant.

One of the central tenants of milk banking is that PDHM support the recipient mother in establishing lactation and successful breastfeeding [11], not to serve as a replacement for high quality lactation support. Our own research in Toronto suggest that owing to several factors related to preterm birth, ∼70% of VLBW infants require supplemental PDHM [6,12]. Many infants in our trials required supplemental PDHM only as a short-term bridge, but for many others, PDHM makes up a significant proportion of their total enteral intake. For the latter group infants, the impact of quality control procedures in milk banking to optimize nutrient content become paramount alongside traditional concerns of microbiological safety. Procedures such as freezing, thawing, container changes, using mature compared with early milk, and pasteurization can variously affect the energy and nutrient content of PDHM compared with fresh maternal milk [13]. Reports of slower in-hospital growth among PDHM-fed VLBW infants due to the aforementioned impacts on milk nutrient composition are well known [7]. Analysis of human milk macronutrient and energy composition is 1 strategy to characterize and improve the nutritional quality of raw and processed milk through quality improvement [11]. Provision of reliable nutritional information on dispensed PDHM labels could be a useful resource for care providers in order to make informed decisions about nutrient fortification. However, unlike the dairy industry, milk banking is at a very early stage of understanding how to implement routine milk analysis.

In this issue, Davis and Perrin [14] summarize findings from their scoping review of peer-reviewed English language publications with the principal aim to assess the impact of Holder pasteurization on the fat concentration of human milk. Fat is known to be the most variable of the macronutrients in human milk, and the literature is mixed on the impact of pasteurization on fat content. A secondary aim of the review was to understand how mixing of milk at the time of sample collection for analysis may have affected the results of studies examining the impact of pasteurization on fat content. Unlike cow milk, human milk is not homogenized during PDHM processing. A “high” degree of mixing in the scoping review was defined as use of a mechanical mixing instrument such as an ultrasonic homogenizer or vortexer; whereas “moderate” mixing included nonmechanical mixing (e.g., inversion). In contrast, a “low” degree of mixing was defined as either the mixing procedures were not specified (e.g., “the pools were mixed”) and, unlike “high” and “moderate” mixing, temperature information during mixing was not provided. Of the 26 studies identified, 10 studies provided information on mixing protocols, whereas 16 did not. Of these 16 studies, half observed a change of ≥5% in fat content between prepasteurization and postpasteurization and this ranged from −28.6% to +19.4%. In contrast, in studies where authors reported on any amount of mixing (low, moderate, or high), prepasteurization and postpasteurization fat values ranged from −6% to 0.0%, likely within measurement error in my estimation. The authors concluded from their scoping review, correctly in my view, that pasteurization per se does not affect the fat concentration of human milk. However, incorporation of mixing with the intent to obtain a representative sample may significantly influence prepasteurization and postpasteurization fat content differences or lack thereof. These findings underscore the need for future research to facilitate development of guidelines on milk sampling through donor human milk processing. They also underscore, in my view, some level of uncertainty of the robustness of the values presently provided on all PDHM labels.

In 2023, HMBANA reported that its 33 member banks (United States and Canada) dispensed nearly 10 million ounces of PDHM to 1500 hospitals [15]. Capacity of many banks now exceeds the volume of PDHM required to serve as a partial or sole source of nutrition for NEC reduction among VLBW infants. Given the capacity of milk banks, increased awareness of the benefits of breastfeeding, and known retention of many important bioactive components in PDHM, supplemental PDHM is being used for other clinical indications (e.g., malabsorption) in some centers. Additionally, supplemental PDHM is provided in some well-baby units and is available for purchase after discharge. Unlike for NEC prevention in VLBW infants, however, evidence that these practices improve infant outcomes is limited [16]. Nonetheless, use of PDHM as a predominant source of nutrition for these expanded applications necessitates both the provision of PDHM of the highest nutritional quality and an understanding of the nutrient composition of PDHM to be shared with health care providers caring for these infants. Evidence-based approaches for sampling and analysis of human milk are critical, as is the availability and regulatory approval of suitable tools for routine human milk analysis in milk banks and in the clinical setting. Support of innovations to allow simplified low-cost technologies to assess the nutritional quality of milk through processing in low-resource settings is necessary [11].

## Author contributions

The sole author was responsible for all aspects of this manuscript.

## Conflict of interest

The authors report no conflicts of interest.
